# Integrated transcriptomic and metabolomic analysis reveals zinc oxide nanoparticles associated modulation of salt stress responses in *Glycyrrhiza uralensis*

**DOI:** 10.3389/fpls.2026.1767899

**Published:** 2026-03-03

**Authors:** Shangtao Wang, Shuang Liang, Shurui Zhang, Siyu Ma, Yaping Li, Yifan Yan, Gonghao Xu, Chenghao Zhu, Zhirong Sun

**Affiliations:** 1 School of Chinese Materia Medica, Beijing University of Chinese Medicine, Beijing, China; 2 Luxi-ratio Ltd., Taipei, Taiwan; 3 Guangxi Institute of Botany, Chinese Academy of Sciences, Guilin, China

**Keywords:** *Glycyrrhiza uralensis*, metabolome, salt stress, transcriptome, zinc oxide nanoparticles

## Abstract

The depletion of wild *Glycyrrhiza* resources has made artificial cultivation essential; however, soil salinization severely limits the yield and quality of *G. uralensis*. Zinc oxide nanoparticles (ZnO NPs) exhibit promising potential in phytoremediation, yet their regulatory mechanisms under salt stress remain unclear. In this study, *Glycyrrhiza uralensis* was used to investigate the effects of salt stress and the modulating effects of ZnO NPs. Our results showed that ZnO NPs application enhanced salt accumulation in *Glycyrrhiza uralensis* while maintaining photosynthetic performance and root growth. Integrated transcriptomic and metabolomic analyses revealed that salt stress disrupted phenylpropanoid and flavonoid biosynthesis pathways, leading to detectable alterations in root metabolic profiles. Moreover, ZnO NPs modulated root metabolite accumulation and related gene expression, which further affected root metabolites and activated plant defense systems, contributing to enhanced root system adaptation and overall plant resilience under salt stress. These findings suggest that ZnO NPs treatment is associated with improved salt stress performance in *G. uralensis* under the present experimental conditions. This study provides insights into the potential application of ZnO nanoparticles in improving salt tolerance, thereby offering a promising strategy for the sustainable cultivation of *Glycyrrhiza uralensis* in saline-alkali soils.

## 1 Introduction

Soil salinization is widely recognized as one of the major abiotic constraints limiting plant growth and agricultural productivity (Daliakopoulos et al., 2016; Kumar et al., 2020). It is estimated that more than one billion hectares of land worldwide are currently affected by salinity, and this problem continues to intensify due to climate change, inappropriate irrigation practices, and secondary salinization (Shabala et al., 2015). Excessive salt accumulation in soils restricts plant water uptake, disrupts cellular ionic homeostasis, and induces oxidative stress, ultimately leading to growth inhibition and yield reduction (Zhang et al., 2023). Therefore, enhancing plant growth and adaptive capacity under saline conditions is of great significance for the sustainable utilization of saline–alkali land resources.

Cultivating economically valuable plant species in salinized soils represents a practical and feasible strategy to mitigate the adverse effects of soil salinity (Van Zelm et al., 2020). *Glycyrrhiza uralensis* Fisch., a perennial leguminous medicinal plant, is widely used in the pharmaceutical, food, and cosmetic industries. Its roots and rhizomes are rich in bioactive compounds, particularly triterpenoid saponins and flavonoids, which serve as key ingredients in numerous traditional and modern formulations (Zhang and Ye, 2009). With increasing market demand and the gradual depletion of wild resources, cultivated *G. uralensis* has become the primary source of supply (Dong et al., 2024). However, the species exhibits limited tolerance to salt stress at the seedling stage, often resulting in reduced survival rates, unstable yield, and compromised quality in salinized soils (Li et al., 2016). Improving salt tolerance is therefore a critical prerequisite for achieving stable and sustainable production of *G. uralensis* under saline conditions.

In recent years, nanotechnology has attracted increasing attention in agricultural research and is considered a promising approach for enhancing plant tolerance to adverse environmental conditions (Rastogi et al., 2019; Hofmann et al., 2020). Zinc is an essential micronutrient for plants, playing crucial roles in enzymatic reactions, regulation of photosynthesis, and antioxidant defense (Stanton et al., 2022; Kaur et al., 2023). Zinc oxide nanoparticles (ZnO NPs), characterized by their small particle size, large specific surface area, and high bioavailability, have been proposed as an alternative form of zinc supplementation (Landa et al., 2015; Caldelas and Weiss, 2017). Previous studies have demonstrated that ZnO NPs can improve growth performance and alleviated salt-induced changes in various crop species (Sun et al., 2020; El-Badri et al., 2021; Alghamdi et al., 2022). However, research on their application in medicinal plants remains limited, and the regulatory effects of ZnO NPs on *G. uralensis* under salt stress have not yet been systematically elucidated. It should be noted that ZnO NPs may exert effects through both nanoparticle-specific properties and the release of Zn²^+^ ions. This study focuses on evaluating the comprehensive effects of ZnO NP application on *G. uralensis* under salt stress, acknowledging the potential contribution of both forms.

Plant salt tolerance is a complex trait entailing coordinated physiological and molecular adaptations. The emergence of high-throughput omics technologies has markedly advanced our systems-level understanding of these adaptive mechanisms. Integrated transcriptomic and metabolomic approaches, in particular, enable the simultaneous profiling of dynamic gene expression and metabolite accumulation, offering profound insights into the molecular basis of salinity adaptation (Gao et al., 2022; Han et al., 2025).

Although accumulating evidence suggests that zinc oxide nanoparticles can improve plant performance under saline conditions, the underlying regulatory processes, particularly in medicinal plant species, remain insufficiently understood. Here, we hypothesized that ZnO NPs treatment would be associated with modulation of salt-induced responses in *Glycyrrhiza uralensis* primarily through the selective modulation of antioxidant-related pathways and associated secondary metabolism, rather than through a general stimulation of growth-related processes. Specifically, we propose that ZnO NPs preferentially influence ROS-scavenging capacity and phenylpropanoid- and flavonoid-related metabolic pathways under salt stress. To test this hypothesis, we integrated physiological measurements with transcriptomic and metabolomic analyses to (i) quantify ZnO NPs–mediated changes in growth and stress-related physiological traits under salinity and (ii) identify transcriptional and metabolic pathways specifically associated with ZnO NPs–regulated salt stress responses.

## 2 Materials and methods

### 2.1 Characterization of ZnO nanoparticles

A total of 0.1 mL of ZnO nanoparticles (ZnO NPs) suspension was dropped onto copper grids for transmission electron microscopy (TEM) observation (FEI Talos F200X, Thermo Fisher Scientific, USA). After air-drying, images were captured and particle size distribution was analyzed using ImageJ software to generate a particle size distribution histogram, and ZnO NPs are in a stable state in solution (**Supplementary Figure 1**).

### 2.2 Plant materials and treatments

Seeds of *Glycyrrhiza uralensis* Fisch. were collected from Yanchi County, Ningxia, China. To remove surface contaminants, the seeds were thoroughly rinsed with ultrapure water three times, immersed in 75% (v/v) ethanol for 30 s, and subsequently treated with 5.0% sodium hypochlorite solution for 10 min. After sterilization, seeds were washed three additional times with ultrapure water. The disinfected seeds were placed in Petri dishes for germination. When the seedlings reached approximately 2 cm in height, uniform individuals were transferred into plastic pots filled with farmland soil at a density of 20 plants per pot. Plants were maintained under controlled environmental conditions with a 14 h light (24 °C)/10 h dark (16 °C) cycle and relative humidity maintained at 60%.

Based on preliminary experiments, 160 mM NaCl was selected as the salinity level and 40 mg·L^-^¹ as the ZnO NPs concentration. Four treatments were established: (1) CK (control, no NaCl and ZnO NPs); (2) S (160 mM NaCl); (3) CK + ZnO NPs (40 mg·L^-^¹ ZnO NPs); and (4) S + ZnO NPs (160 mM NaCl + 40 mg·L^-^¹ ZnO NPs). The first treatment was applied when the first true leaf emerged, and 200 mL of the corresponding solution was evenly applied to each pot. Treatments were repeated every three days for a total of ten applications. Control plants were irrigated with the same volume of ultrapure water. The experiment was conducted using a completely randomized design. Each treatment consisted of three independent biological replicates, with each replicate comprising 20 seedlings grown in a single pot. All physiological and biochemical measurements were performed using independent biological samples, and technical replicates were averaged prior to statistical analysis.

After one month of treatment, plant samples were collected. Different tissues were harvested to evaluate the effects of salinity and ZnO NPs. Root samples were immediately frozen in liquid nitrogen and stored at −80 °C for transcriptomic and metabolomic analyses.

### 2.3 Determination of physiological parameters

Physiological traits were determined using five fully expanded healthy leaves per treatment. Net photosynthetic rate (Pn), transpiration rate (Tr), stomatal conductance (Gs), and intercellular CO_2_ concentration (Ci) were measured with a portable photosynthesis system (Yaxin-1105, Beijing, China) following previously reported protocols (Tirani et al., 2013; Fan et al., 2021). Measurements were performed between 09:00 and 11:00 under a controlled CO_2_ concentration of 400 μmol·mol^-^¹ and an airflow rate of 0.6 m·min^-^¹. Malondialdehyde (MDA) levels were quantified using the thiobarbituric acid (TBA) method to evaluate lipid peroxidation. Enzymatic activities of superoxide dismutase (SOD), and peroxidase (POD) in leaf tissues were determined using commercial assay kits (Jiangsu Edison Biotechnology Co., Ltd., China) in accordance with the manufacturer’s instructions (Kubis, 2005; Jambunathan, 2010; Kim et al., 2012). The concentrations of sodium (Na^+^) and zinc (Zn^2+^) in plant tissues were measured using inductively coupled plasma mass spectrometry (ICP–MS). For elemental analysis, 0.1 g of dried leaf and root samples was digested in 2.5 mol·L^-^¹ nitric acid, and the resulting solutions were subjected to analysis after appropriate dilution.

### 2.4 Metabolomic analysis

Fresh root samples were freeze-dried, finely ground into powder, and extracted using 70% (v/v) methanol solution. The extracts were passed through 0.22 μm membrane filters and transferred into autosampler vials for analysis. Metabolite profiling was conducted using an ultra-performance liquid chromatography system coupled with tandem mass spectrometry (UPLC–ESI–MS/MS; ExionLC™ AD, Shanghai, China). Chromatographic separation was carried out on a Kinetex C18 column (2.1 mm × 100 mm, 2.6 μm). The mobile phase consisted of solvent A (0.01% acetic acid in water) and solvent B (50% acetonitrile/isopropanol). The column temperature was maintained at 25 °C, while the autosampler temperature was set at 4 °C. A sample volume of 2 μL was injected at a flow rate of 0.3 mL·min^-^¹. Mass spectrometric detection was performed on a QTrap 6500+ system (Sciex Technologies). The electrospray ionization parameters were as follows: ion spray voltage of 5500 V (positive mode) and −4500 V (negative mode), ion source temperature of 400 °C, curtain gas at 35 psi, and ion source gas 1 at 50 psi.

LC–MS data acquisition and analysis were performed by Baipu Biotechnology Co., Ltd. (Shanghai, China). Differential metabolites were screened based on the criteria of fold change ≤ 0.5 or ≥ 2 and adjusted p-value < 0.05. Identified differential metabolites were mapped to the KEGG database for pathway enrichment analysis.

### 2.5 Transcriptomic analysis

Total RNA was isolated from licorice root tissues using the RNeasy Pure Plant Kit (DP432, Tiangen, China). RNA integrity was evaluated by 1% agarose gel electrophoresis, and purity and concentration were assessed using a NanoPhotometer spectrophotometer (IMPLEN, USA).

Double-stranded cDNA libraries were prepared using the RNA Library Prep Kit (Tiangen, Beijing, China), and high-throughput sequencing was performed on the Illumina HiSeq platform by Baiqu Biotechnology Co., Ltd. (Shanghai, China). Raw sequencing reads were quality-filtered to eliminate low-quality reads, and GC content and sequencing error rate were analyzed to ensure data reliability. Clean reads were assembled *de novo* using Trinity software to generate reference transcripts. Unigenes were obtained through hierarchical clustering using Corset. Differentially expressed genes (DEGs) were identified using the DESeq2 package with the thresholds of fold change ≤ 0.5 or ≥ 2 and adjusted p-value < 0.05. Functional annotation was performed based on Gene Ontology (GO), and Kyoto Encyclopedia of Genes and Genomes (KEGG) pathway enrichment analyses were conducted. Weighted gene co-expression network analysis (WGCNA) was carried out using the WGCNA package implemented in R software (v3.3.0) to identify gene modules associated with different treatments.

### 2.6 Statistical analysis

All statistical analyses were conducted using SPSS version 22.0 (IBM Corp., USA). Differences among treatment groups were assessed by one-way analysis of variance (ANOVA) followed by Tukey’s multiple comparison test. A significance level of p < 0.05 was applied. Visualization of transcriptomic and metabolomic datasets, including Venn diagrams, heatmaps, and principal component analysis (PCA) plots, was generated using the OmicShare online platform (www.omicshare.com/tools). Additional figures were produced using GraphPad Prism 10.0 (GraphPad Software, USA) and Adobe Illustrator 2024 (Adobe Inc., USA). The transcriptomic data have been deposited in the NCBI Sequence Read Archive (SRA) under accession number PRJNA1421034.

## 3 Results

### 3.1 Characterization and determination of ZnO NPs

**Figure 1:**
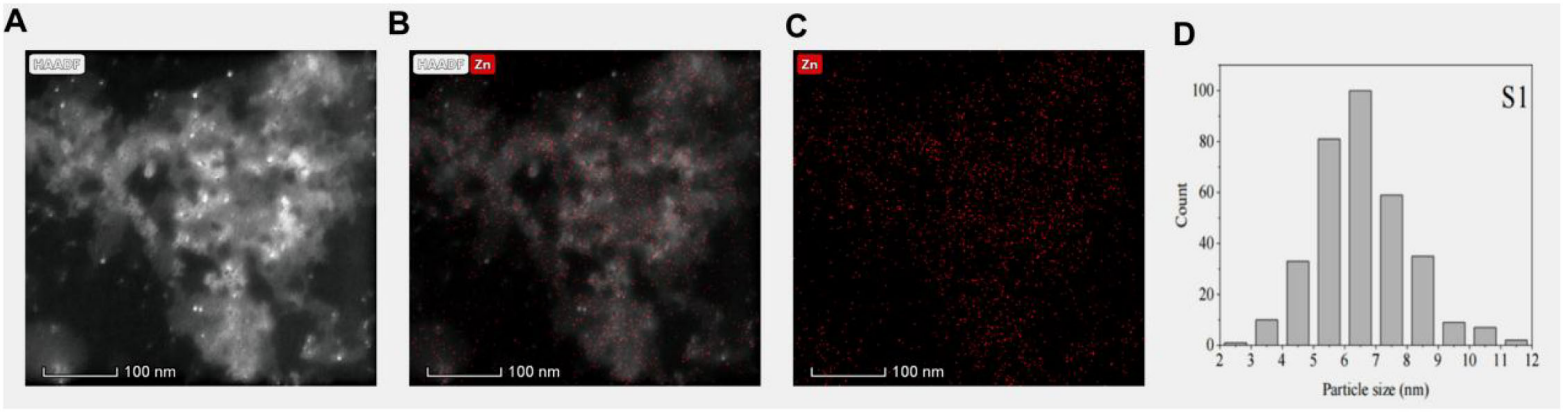
Chemical characterization of ZnO NPs. **(A)** NAADF imaging by transmission electron microscopy. **(B)** Zn imaging by transmission electron microscopy. **(C)** combined imaging by transmission electron microscopy. **(D)** Histogram of ZnO NPs size distribution.

As demonstrated by the transmission electron microscopy scanning results, the ZnO NPs exhibited a predominant spherical morphology under electron microscope observation (**Figures 1A–C**). The size distribution of the particles ranged from 2–12 nm, with an average and median size of 6.5 nm (**Figure 1D**).

### 3.2 Plant traits

**Figure 2:**
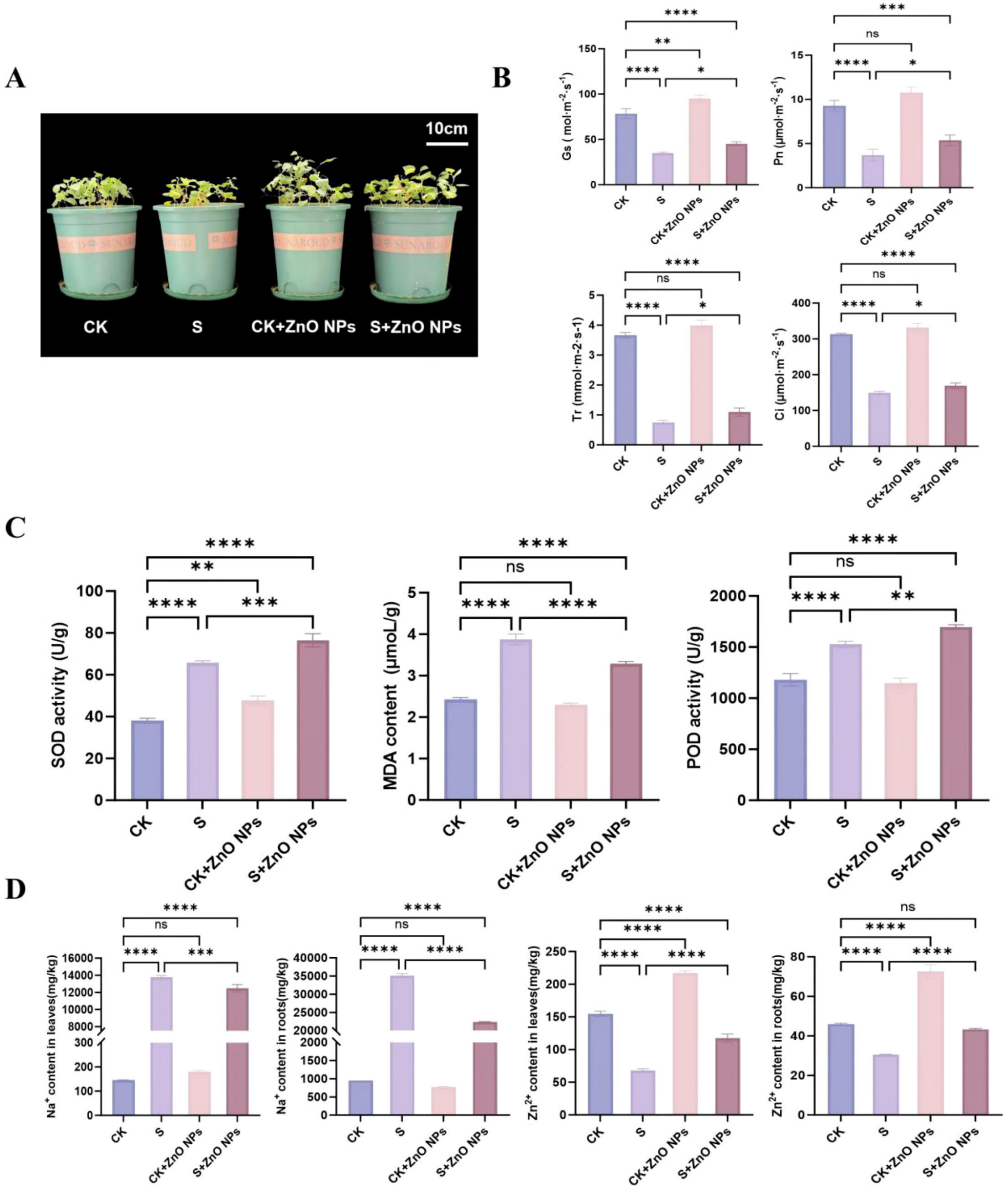
Responses of *Glycyrrhiza uralensis* to salt and ZnO NPs application. **(A)** The morphology of *G. uralensis* under the different treatments. **(B)** Effects on stomatal conductance (Gs, mol m^−2^ s^−1^), net photosynthetic rate (Pn, μmol m^−2^ s^−1^), transpiration rate (Tr, mmol m^−2^ s^−1^), CO_2_ concentration (Ci, μmol m^−2^ s^−1^) of plants treated with salt and ZnO NPs. **(C)** The enzymatic activities of superoxide dismutase (SOD), peroxidase (POD), and Malondialdehyde (MDA) in leaf tissues of *G. uralensis.* **(D)** The content of Na^+^ and Zn^2+^ in roots and leaves of *G. uralensis*.

To assess the effects of salinity and zinc oxide nanoparticles (ZnO NPs) on *G. uralensis*, leaf morphology was examined. Salt treatment caused visible chlorosis at the leaf tips, whereas this symptom was markedly alleviated under combined salt and ZnO NPs exposure (**Figure 2A**), indicating that ZnO NPs promoted plant growth under salt stress. Photosynthetic performance and leaf physiological status were further evaluated using multiple gas-exchange parameters. Exogenous application of ZnO NPs significantly mitigated the salt-induced decreases in stomatal conductance (Gs), net photosynthetic rate (Pn), transpiration rate (Tr), and intercellular CO_2_ concentration (Ci); however, these values remained lower than those observed under control (CK) conditions (**Figure 2B**). In addition, ZnO NPs treatment resulted in increased chlorophyll and carotenoid contents in leaves (**Supplementary Figure 2**), suggesting improved photosynthetic pigment accumulation. Among the antioxidant-related indicators analyzed, ZnO NPs treatment was associated with a significant increase in SOD activity compared with salt stress alone., whereas no significant effects were observed for malondialdehyde (MDA) content or peroxidase (POD) activity. Nevertheless, in the S + ZnO NPs treatment group, SOD activity, as well as MDA and POD levels, were higher than those in the control group (**Figure 2C**). Elemental analysis showed that Na^+^ accumulation in roots was highest under salt stress alone. In contrast, under combined S and ZnO NPs treatment, Na^+^ contents in both leaves and roots declined, while Zn^2+^ contents increased (**Figure 2D**), indicating that ZnO NPs altered ion uptake and distribution in *G. uralensis* seedlings.

### 3.3 Alterations in root metabolism in response to salt stress and ZnO NPs

**Figure 3:**
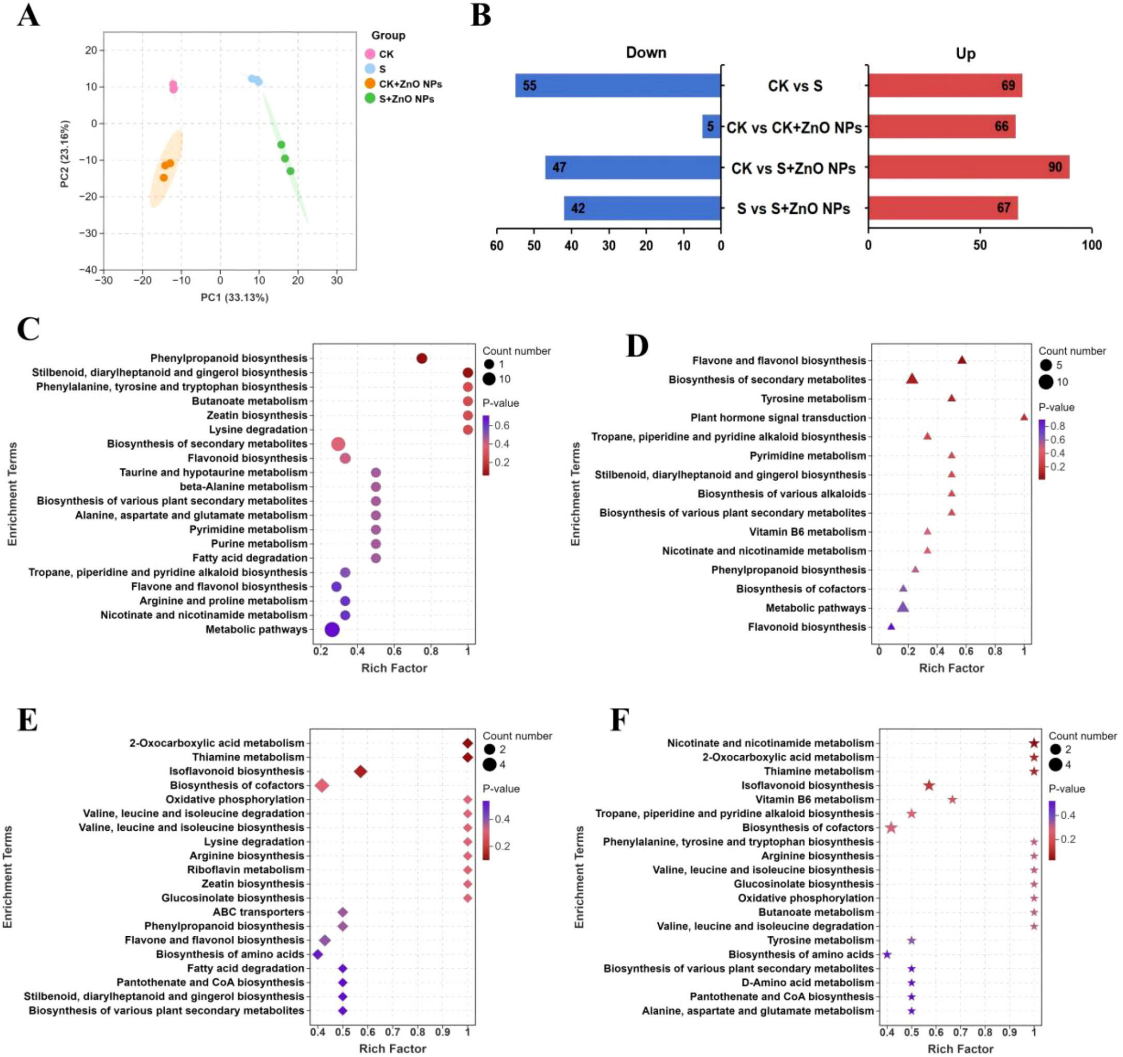
Analysis of metabolomic changes under different treatments. **(A)** Principal component analysis of metabolites identified in each treatment group. **(B)** The number of upregulated and downregulated differentially expressed metabolites (DEMs) in pairwise comparisons. **(C)** KEGG enrichment bubble plot of differential metabolites between CK and M comparison groups. **(D)** KEGG enrichment bubble plot of differential metabolites between CK and CK+ZnO NPs comparison groups. **(E)** KEGG enrichment bubble plot of differential metabolites between CK and M+ZnO NPs comparison groups. **(F)** KEGG enrichment bubble plot of differential metabolites between M and M+ZnO NPs comparison groups.

Metabolomic profiling was performed to elucidate the global metabolic responses of *G. uralensis* roots to salt stress and ZnO NPs. Across all four treatments, a total of 580 metabolites were identified in *G. uralensis* roots. Principal component analysis (PCA) showed that the first two principal components (PC1 and PC2) explained 33.13% and 23.16% of the total variance, respectively. Clear separations were observed between CK samples and those subjected to salt stress and combined salt and ZnO NPs treatments (**Figure 3A**).

A total of 257 differentially accumulated metabolites (DAMs) were identified across the four pairwise comparisons. Based on relative abundance, 124 DAMs were detected in CK vs S, 71 in CK vs CK + ZnO NPs, and 109 in S vs S + ZnO NPs. In addition, 137 DAMs differed significantly between CK and S + ZnO NPs samples (**Figure 3B**). Among these DAMs, 134 were shikimate- and phenylpropanoid-related compounds, 35 were alkaloids, 25 were terpenoids, 19 were amino acids and short peptides, 14 were carbohydrates, 14 were polyketides, 12 were fatty acids, and 4 belonged to other categories (**Supplementary Table 1**).

Comparative analysis revealed pronounced differences in metabolic profiles among CK, S, CK + ZnO NPs, and S + ZnO NPs treatments. In CK vs CK + ZnO NPs, differential accumulation was observed in shikimate and phenylpropanoid-related compounds, alkaloids, carbohydrates, terpenoids, amino acids and short peptides, fatty acids, and polyketides, with only three shikimate-derived metabolites and one carbohydrate being downregulated. Under salt stress, shikimate and phenylpropanoid-related compounds constituted the most enriched class, followed by alkaloids, amino acids and short peptides, terpenoids, and fatty acids. In contrast, the concentrations of 31 shikimate and phenylpropanoid-related metabolites, 6 alkaloids, 5 amino acids and short peptides, 3 terpenoids, 2 fatty acids, and 1 polyketide were reduced under salt exposure. These findings indicate that ZnO NPs and salt treatments exerted distinct effects on the abundance and composition of root metabolites.

In the CK vs S + ZnO NPs comparison, shikimate and phenylpropanoid-related metabolites were the most enriched category, followed by terpenoids, alkaloids, and fatty acids. Among the DAMs, 47 shikimate and phenylpropanoid-related metabolites were upregulated, whereas the downregulated metabolites were mainly associated with shikimate and phenylpropanoid metabolism, terpenoids, alkaloids, carbohydrates, and amino acids and short peptides (**Supplementary Table 1**). These results suggest that metabolites related to the shikimate and phenylpropanoid pathways may play important roles in the beneficial effects of ZnO NPs on *G. uralensis* under salt stress.

To identify key metabolic pathways associated with S, ZnO NPs, and combined treatments, KEGG pathway enrichment analysis was conducted for the top 20 significantly enriched pathways. In the CK vs S comparison, 124 DAMs were significantly enriched in pathways including phenylpropanoid biosynthesis, biosynthesis of secondary metabolites, flavonoid biosynthesis, and biosynthesis of various plant secondary metabolites (**Figure 3C**). In CK vs CK + ZnO NPs, 16 enriched pathways were identified, including biosynthesis of secondary metabolites, alkaloid biosynthesis, plant secondary metabolite biosynthesis, phenylpropanoid biosynthesis, and flavonoid biosynthesis (**Figure 3D**). Notably, phenylpropanoid and flavonoid biosynthesis pathways were significantly enriched in both CK vs S and CK vs CK + ZnO NPs comparisons.

In CK vs S + ZnO NPs, enriched pathways included isoflavonoid biosynthesis, valine, leucine and isoleucine biosynthesis, phenylpropanoid biosynthesis, amino acid biosynthesis, biosynthesis of various plant secondary metabolites, flavonoid biosynthesis, and biosynthesis of secondary metabolites (**Figure 3E**), with most DAMs exhibiting significantly higher concentrations than those in the control group. Furthermore, in the S vs S + ZnO NPs comparison, DAMs were primarily enriched in nicotinate and nicotinamide metabolism, isoflavonoid biosynthesis, phenylalanine, tyrosine and tryptophan biosynthesis, amino acid biosynthesis, plant secondary metabolite biosynthesis, flavonoid biosynthesis, and secondary metabolite biosynthesis (**Figure 3F**).

Collectively, these results indicate that DAMs involved in secondary metabolism, phenylpropanoid biosynthesis, amino acid biosynthesis, and flavonoid biosynthesis may play critical roles in the mitigation of salt stress in *G. uralensis* roots by ZnO NPs.

### 3.4 Salt stress and ZnO NPs application induce transcriptomic reprogramming in *G. uralensis* roots

**Figure 4:**
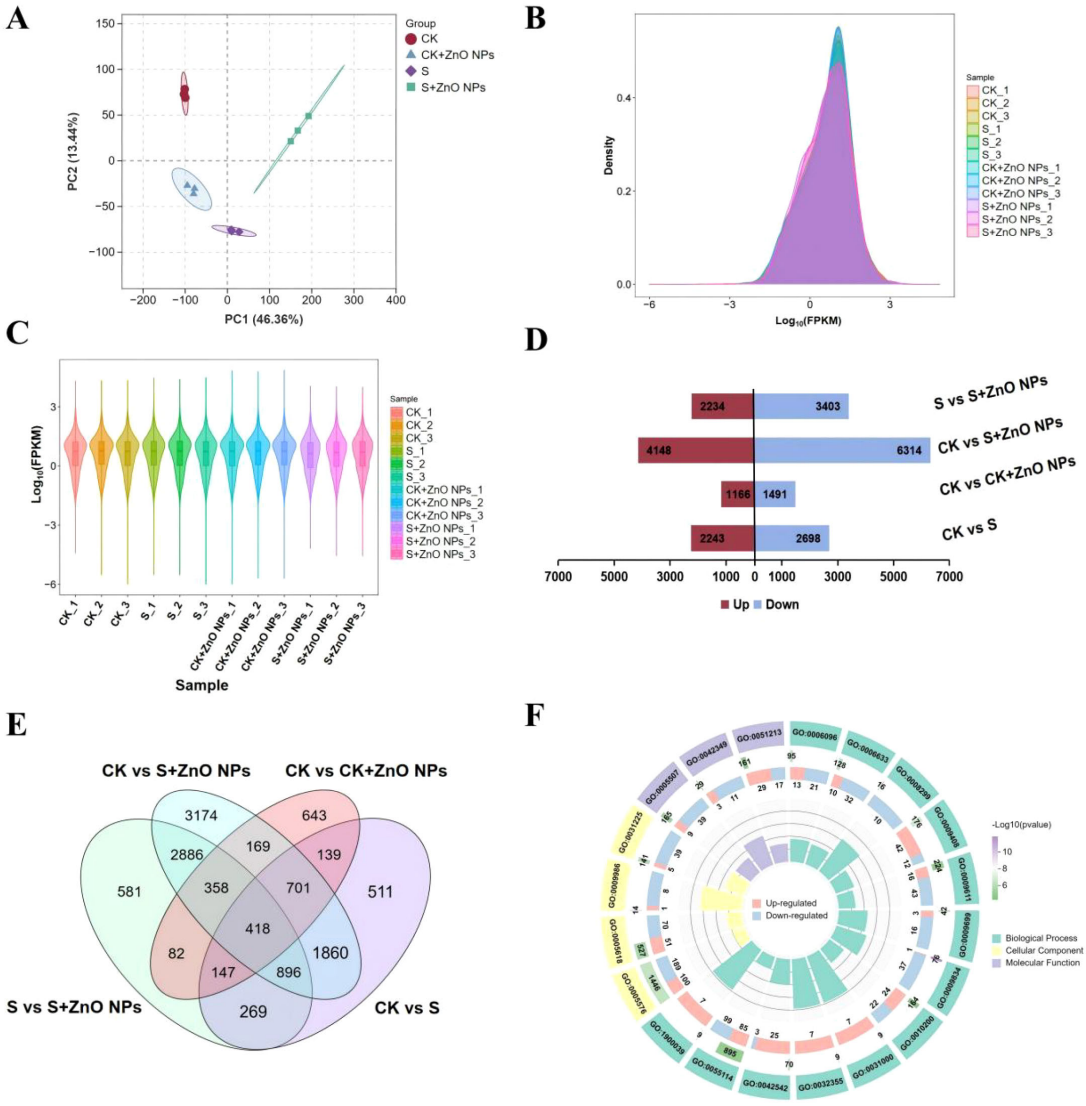
Transcriptional relationship and differentially expressed genes **(DEGs)** between different samples. **(A)** Principal component analysis of expressed genes in the treatment groups. **(B)** The FPKM distribution of different samples. **(C)** Gene expression of different samples. **(D)** The number of upregulated and downregulated genes identified in pairwise comparisons. **(E)** Venn diagram of the number of DEGs identified by comparisons among the CK and treatment groups. **(F)** GO enrichment circle diagram of CK and S comparison groups.

Transcriptomic analysis was conducted to investigate global gene expression profiles of *G. uralensis* roots in response to ZnO NPs and salt stress. A total of twelve root cDNA libraries were constructed and subjected to RNA sequencing. All libraries exhibited Q30 scores exceeding 98.09%, indicating high sequencing quality and reliability (**Supplementary Table 2**). Principal component analysis (PCA) revealed detectable differences in gene expression patterns among treatments (**Figure 4A**), with the twelve samples clustering into four distinct groups. In addition, the distribution of gene expression levels and fragments per kilobase of transcript per million mapped reads (FPKM) values further confirmed the distinct transcriptional responses across the four treatments (**Figures 4B, C**), highlighting treatment-specific transcriptomic profiles.

Across CK vs CK + ZnO NPs, CK vs S, CK vs S + ZnO NPs, and S vs S + ZnO NPs comparisons, a total of 23,697 differentially expressed genes (DEGs) were identified (**Figure 4D**). Venn diagrams illustrated the overlap and uniqueness of DEGs among the four comparison groups (**Figure 4E**). The CK vs S + ZnO NPs comparison yielded the highest number of DEGs, suggesting that combined salt and ZnO NPs treatment exerted the strongest impact on the *G. uralensis* root transcriptome. In contrast, the fewest DEGs were detected in the CK vs CK + ZnO NPs comparison. A total of 4,941 DEGs were identified in CK vs S, reflecting transcriptional adjustments in response to salt stress alone. Moreover, 5,637 DEGs were found between S and S + ZnO NPs, indicating that ZnO NPs substantially altered gene expression under saline conditions. Notably, 418 DEGs were shared across all comparisons and were considered associated candidate genes responsive to ZnO NPs exposure under salt stress (**Figure 4E**).

**Figure 5:**
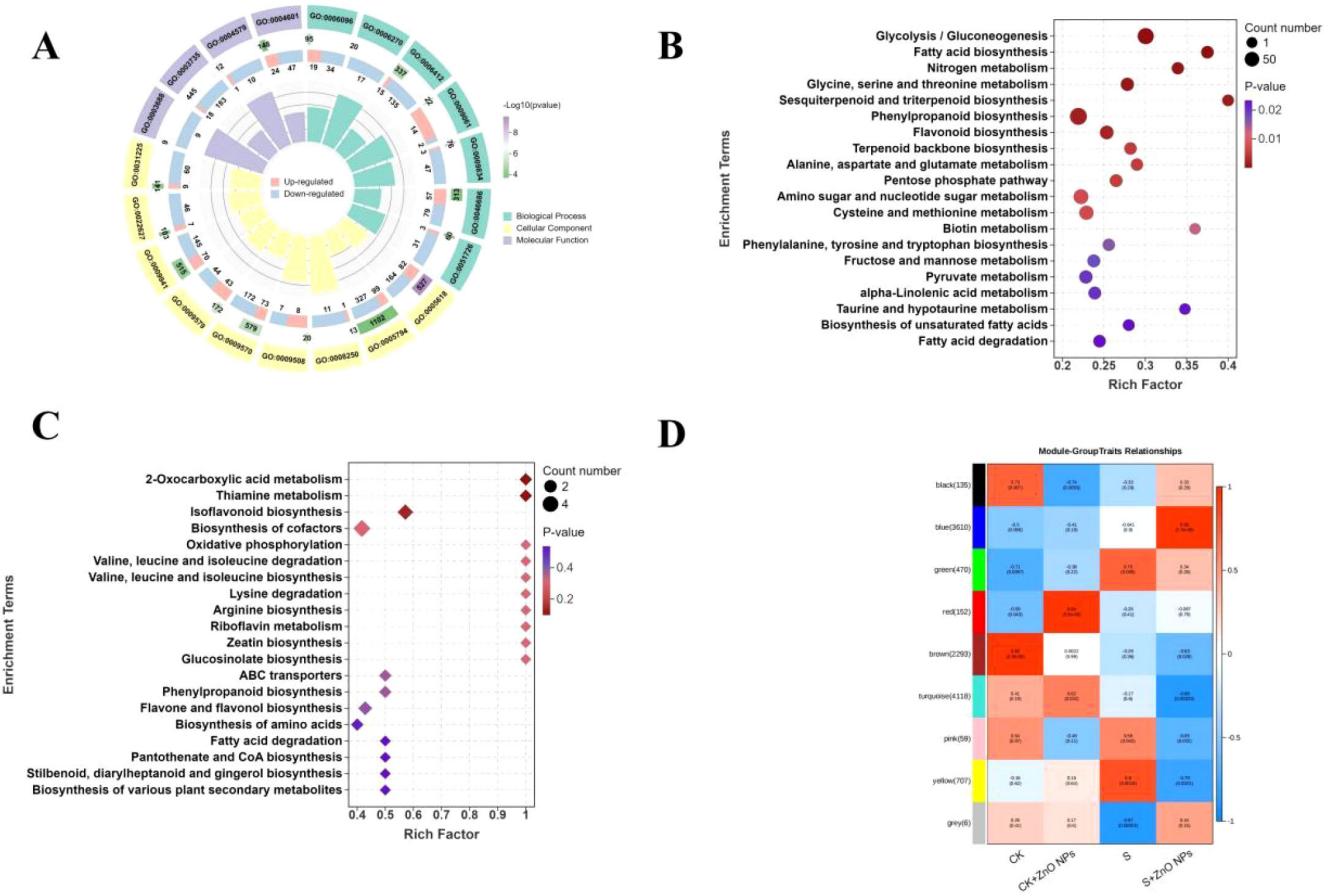
Analysis of differentially expressed genes (DEGs) among different samples. **(A)** GO enrichment circle diagram of CK and S+ZnO NPs comparison groups. **(B)** KEGG enrichment bubble plot of differential genes between CK and M comparison groups. **(C)** KEGG enrichment bubble plot of differential genes between CK and S+ZnO NPs comparison groups. **(D)** Gene expression modules based on weighted gene co-expression network analysis (WGCNA).

Gene Ontology (GO) enrichment analysis revealed that DEGs in CK vs S were significantly associated with biological processes and molecular functions such as plant-type secondary cell wall biogenesis, response to hydrogen peroxide, glycolytic process, phenylpropanoid biosynthetic process, dioxygenase activity, and peroxidase activity, which are closely related to salt stress responses and cellular homeostasis (**Figure 4F**). Specifically, upregulated DEGs were mainly enriched in response to heat, response to hydrogen peroxide, dioxygenase activity, response to chitin, and response to caffeine, whereas downregulated DEGs were primarily associated with plant-type secondary cell wall biogenesis, extracellular region, fatty acid biosynthetic process, and glycolytic process (**Figure 4F**). GO enrichment analysis of DEGs in CK vs S + ZnO NPs showed that genes associated with plastid chromosome, anaerobic respiration, photosynthesis, and trehalose biosynthetic process were significantly upregulated. In contrast, DEGs related to cell wall, plant-type secondary cell wall biogenesis, structural constituent of ribosome, and glycolytic process were downregulated (**Figure 5A**), which may contribute to enhanced salt tolerance in *G. uralensis*.

KEGG pathway enrichment analysis indicated that DEGs in all comparison groups were significantly enriched in the sesquiterpenoid and triterpenoid biosynthesis pathway. In addition, DEGs in CK vs S were enriched in multiple pathways, including fatty acid biosynthesis, nitrogen metabolism, phenylpropanoid biosynthesis, and flavonoid biosynthesis (**Figure 5B**). In the CK vs S + ZnO NPs comparison, enriched pathways included glycine, serine and threonine metabolism, isoflavonoid biosynthesis, citrate cycle (TCA cycle), and starch and sucrose metabolism (**Figure 5C**).

Weighted gene co-expression network analysis (WGCNA) identified nine distinct gene modules (**Supplementary Figure 3**). Strong correlations were observed between specific modules and treatments. As shown in **Figures 5D**, the blue module was positively correlated with the S + ZnO NPs treatment, the green and yellow modules showed positive associations with salt-treated samples, and the red module was positively correlated with ZnO NPs treatment alone. KEGG analysis of genes in the blue module revealed significant enrichment in plant hormone signal transduction, glycolysis/gluconeogenesis, amino acid metabolism, flavonoid biosynthesis, phenylpropanoid biosynthesis, and carbohydrate metabolism (**Supplementary Table 3**). The upregulation of these metabolism-related genes is consistent with the metabolomic findings and suggests that ZnO NPs alleviate salt stress in *G. uralensis* roots by modulating associated metabolic pathways.

### 3.5 Integrated analysis of transcriptomic and metabolomic data in associated pathways

To further narrow down the associated pathways involved in the response to salt stress and to comprehensively elucidate the mitigating effects induced by ZnO NPs, an integrated analysis of transcriptomic and metabolomic data was performed. The results showed that significantly enriched pathways at both the transcript and metabolite levels were consistently detected only in the CK vs S, CK vs S + ZnO NPs, and S vs S + ZnO NPs comparisons, whereas relatively few shared pathways were identified in the CK vs CK + ZnO NPs comparison (**Supplementary Figure 3**). These findings indicate that transcripts and their associated metabolites exhibited similar functional responses and expression trends under different treatments. Ultimately, the phenylpropanoid biosynthesis and flavonoid biosynthesis pathways were identified as the major common pathways associated with both differentially expressed genes (DEGs) and differentially accumulated metabolites (DAMs).

**Figure 6:**
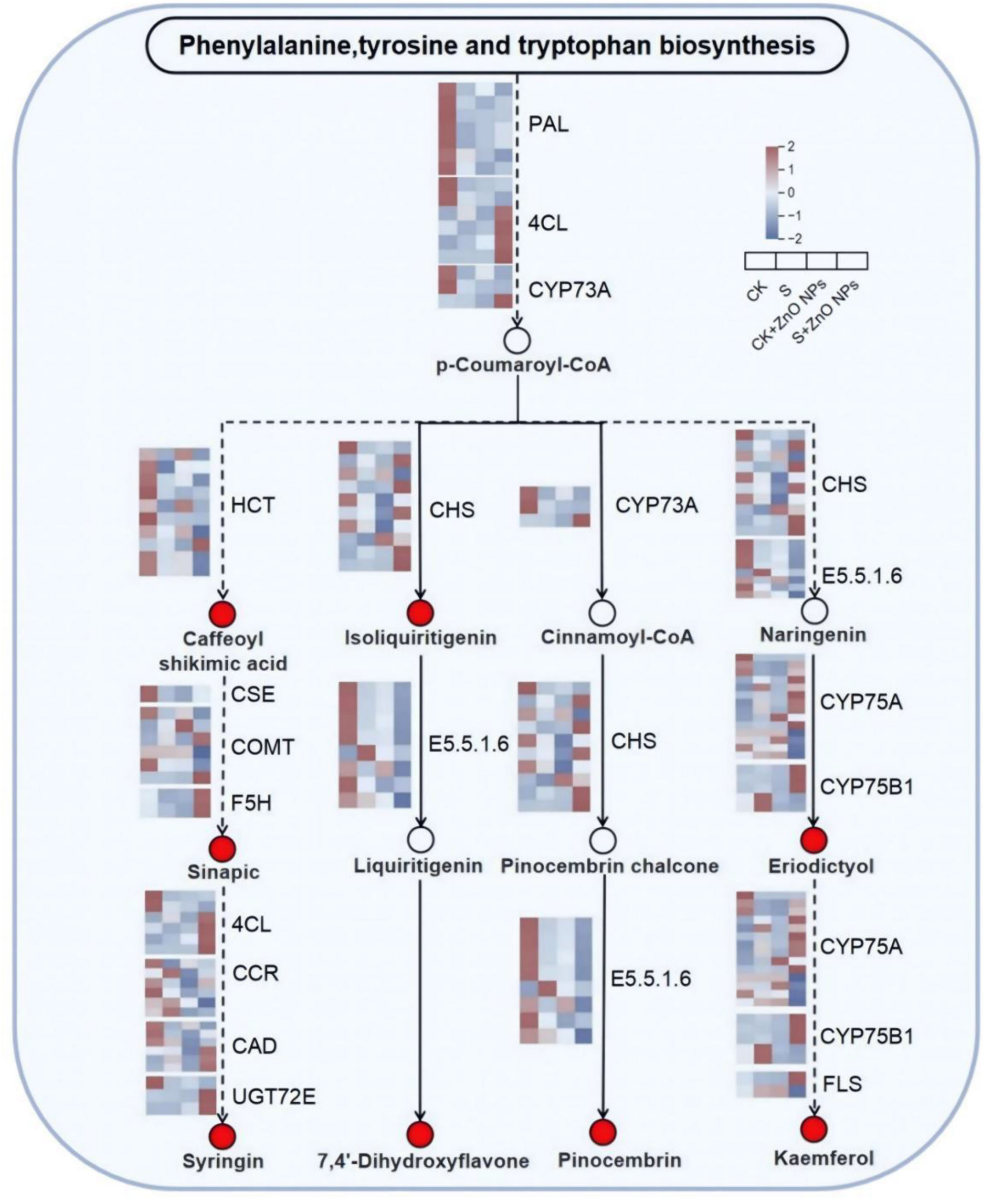
Schematic models of phenylpropane biosynthesis and flavonoid biosynthesis. heatmaps show relative expression values of each DEG. Each row of the heatmap represents one gene and metabolite, and each column represents a single experimental group (from left to right, CK, S, CK + ZnO NPs and S+ZnO NPs groups).The red circle represents differential metabolites. PAL (phenylalanine ammonia-lyase [EC:4.3.1.24]); CYP73A(trans-cinnamate 4-monooxygenase [EC:1.14.14.91]); 4CL(4-coumarate--CoA ligase [EC:6.2.1.12]); HCT(shikimate O-hydroxycinnamoyltransferase [EC:2.3.1.133]); CSE(caffeoylshikimate esterase [EC:3.1.1.-]); COMT(caffeic acid 3-O-methyltransferase/acetylserotonin O-methyltransferase [EC:2.1.1.68 2.1.1.4]); F5H(ferulate-5-hydroxylase [EC:1.14.-.-]) CCR(cinnamoyl-CoA reductase [EC:1.2.1.44]); CAD(cinnamyl-alcohol dehydrogenase [EC:1.1.1.195]) UGT72E(coniferyl-alcohol glucosyltransferase [EC:2.4.1.111]); CHS(chalcone synthase [EC:2.3.1.74]) E5.5.1.6(chalcone isomerase [EC:5.5.1.6]); CYP75A(flavonoid 3’,5’-hydroxylase [EC:1.14.14.81]); CYP75B1(flavonoid 3’-monooxygenase [EC:1.14.14.82]); FLS(flavonol synthase [EC:1.14.20.6]).

As shown in **Figure 6**, a total of 16 DEGs and 8 DAMs involved in the phenylpropanoid and flavonoid biosynthesis pathways were identified. The concentrations of five DAMs—4’,7-dihydroxyflavone, sinapic acid, kaempferol, eleutheroside, and 5-caffeoylshikimic acid—were increased in the S, CK + ZnO NPs, and S + ZnO NPs groups. In contrast, the levels of kaempferol, 5,7-dihydroxyflavanone, and isoscutellarein were higher in the CK group, whereas trihydroxychalcone and 7-dihydroxyflavanone were downregulated under salt treatment but significantly upregulated in the S + ZnO NPs group.

At the transcriptional level, the expression levels of *PAL*, *HCT*, *SCE*, *CCR*, and *E5.5.1.6* were downregulated in the S + ZnO NPs group compared with the control, whereas *4CL*, *F5H*, *UGT72E*, *CYP75A*, *CYP75B*, and *FLS* were significantly upregulated, indicating that ZnO NPs treatment was associated with transcriptional reprogramming consistent with enhanced flavonoid biosynthesis under salt stress.

## 4 Discussion

Salt stress severely constrains plant growth by disrupting water relations, ionic balance, and redox homeostasis (Sofy et al., 2020). Consistent with previous reports on the salt sensitivity of *Glycyrrhiza uralensis* at the seedling stage, saline treatment in this study resulted in detectable growth inhibition and physiological disturbance (Faizan et al., 2021; Adil et al., 2022). The application of ZnO NPs partially alleviated these effects, as indicated by moderate improvements in growth-related traits and selected physiological parameters under salt stress. Importantly, these changes reflect a mitigation of salt-induced impairment rather than an overall stimulation of growth, underscoring a stress-modulatory role of ZnO NPs. In *G. uralensis*, salt stress significantly reduced photosynthetic performance, whereas under combined salt and ZnO NPs treatment, net photosynthetic rate (Pn), stomatal conductance (Gs), intercellular CO_2_ concentration (Ci), and transpiration rate (Tr) were significantly enhanced. These findings indicate that ZnO NPs play an active role in restoring photosynthetic activity in *G. uralensis*. Similar improvements in photosynthetic characteristics following ZnO NPs treatment have been reported in lettuce (Xu et al., 2018). An interesting observation in this study was that SOD activity responded more sensitively to ZnO NPs application under salt stress than POD activity or MDA content. SOD represents the first line of enzymatic defense against oxidative stress by catalyzing the dismutation of superoxide radicals, whereas POD participates in downstream hydrogen peroxide scavenging. The preferential increase in SOD activity may therefore reflect an early or primary adjustment of the antioxidant system rather than a comprehensive activation of all ROS-scavenging enzymes. Importantly, the absence of pronounced changes in POD activity and MDA levels suggests that ZnO NPs treatment may mitigate oxidative stress without inducing excessive lipid peroxidation, rather than broadly amplifying antioxidant enzyme activities.

One of the enriched strategies by which plants acquire salt tolerance is the maintenance of intracellular ion homeostasis under saline conditions. During the uptake of water and nutrients, *G. uralensis* roots inevitably absorb excessive amounts of salt ions, leading to ion imbalance within cells. In this study, salt stress significantly increased Na^+^ accumulation in both roots and leaves. However, ZnO NPs application reduced Na^+^ absorption in these tissues. This effect may be attributed to enhanced zinc availability in the growth environment, which increased Zn accumulation in plant tissues and in turn inhibited Na^+^ uptake by roots and restricted Na^+^ translocation from xylem vessels to mesophyll cells (Zhou et al., 2019). Consequently, ZnO NPs treatment was associated with improved physiological performance under saline conditions.

The response of *G. uralensis* roots to salt stress involves complex mitigation and adaptation mechanisms that likely include the coordinated regulation of multiple functional genes and metabolites. In this study, a large number of genes and metabolites in *G. uralensis* roots exhibited differential expression and accumulation under salt stress and combined salt and ZnO NPs treatments, indicating their involvement in stress alleviation processes. Similar observations have been reported previously, demonstrating that salt stress disrupts the balance between reactive oxygen species (ROS) and antioxidant systems, leading to oxidative damage to cellular components (Hossain and Dietz, 2016; Dumanovic et al., 2021). ROS accumulation under saline conditions may result from complex indirect mechanisms, such as inhibition of enzyme activities. Under these circumstances, plants require sufficient energy and nutrients to counteract oxidative stress (Hossain and Dietz, 2016) (Miller et al., 2010). It has been reported that zinc oxide nanoparticles improve nutrient uptake and reduce oxidative stress (Rizwan et al., 2019). Accordingly, our results indicate that ZnO NPs exposure alleviates the adverse effects of salt stress, which may explain the enhanced growth observed in *G. uralensis*.

Soil application of ZnO NPs can modify antioxidant properties and promote the biosynthesis of specific metabolites in plant roots (Faizan et al., 2021) (García-Gómez et al., 2017). In the present study, numerous functional genes and metabolites in *G. uralensis* roots responded to both salt stress and combined S + ZnO NPs treatments, suggesting that they participate in stress alleviation mechanisms. In addition, salt stress strongly influenced the expression of genes associated with nutrient uptake and transport as well as the accumulation of related metabolites. Our results show that ZnO NPs play a crucial role in promoting *G. uralensis* growth under salt exposure by modulating the accumulation of shikimates and phenylpropanoids, alkaloids, amino acids and peptides, and carbohydrates. Specifically, in the CK vs. S comparison, 71 shikimate- and phenylpropanoid-related compounds were affected by salt exposure; in the CK vs. CK + ZnO NPs comparison, 65 such compounds were identified; and in the S vs. S + ZnO NPs comparison, 47 were significantly altered. These results indicate that both salt stress and ZnO NPs application ultimately reshape the shikimate and phenylpropanoid metabolic pathways.

High salinity shifts *G. uralensis* from a normal growth state toward stress-responsive and defensive metabolism. This transition is likely associated with enhanced gene expression and metabolic reprogramming (Landa et al., 2015; Sun et al., 2020). Integrative transcriptomic and metabolomic analyses identified associated pathways shared by differentially expressed genes (DEGs) and differentially accumulated metabolites (DEMs), including phenylalanine, tyrosine, and tryptophan biosynthesis, phenylpropanoid biosynthesis, and flavonoid biosynthesis. As major products of the phenylpropanoid pathway, caffeoyl shikimate, sinapic acid, and syringin have been widely reported as stress-responsive metabolites in multiple transcriptomic and metabolomic studies (Li et al., 2017; Geng et al., 2020; Zhang et al., 2021), and they were significantly regulated by salt stress and ZnO NPs treatment in our study. Notably, salt stress negatively regulated the transcription of *4CL*, *F5H*, *CAD*, and *UGT72E*, whereas these genes were upregulated under S + ZnO NPs conditions, suggesting that activation of enzyme activities under ZnO NPs treatment may contribute to alleviating salt stress.

Importantly, p-coumaroyl-CoA acts as a metabolic intermediate linking phenylpropanoid biosynthesis and flavonoid biosynthesis. Under S + ZnO NPs treatment, ZnO NPs application significantly altered the expression of key flavonoid biosynthesis genes, including *CYP73A*, *CHS*, *CYP75A*, *CYP75B*, and *FLS*, which are involved in the biosynthesis of several flavonoids, such as 4′,7-dihydroxyflavone, trihydroxychalcone, and 5,7-dihydroxyflavanone. Therefore, integrative analysis of transcriptomic and metabolomic data suggests that ZnO NPs alleviated salt-induced changes by regulating metabolite accumulation and gene expression in roots, thereby strengthening defense systems and improving stress resilience.

Overall, by integrating physiological, transcriptomic, and metabolomic analyses, this study provides a multi-level perspective on ZnO NPs–mediated modulation of salt stress responses in *G. uralensis*. Rather than acting as general growth enhancers, ZnO NPs appear to fine-tune antioxidant defenses and stress-associated regulatory networks. These findings offer a cautious but informative framework for evaluating the potential application of ZnO NPs in medicinal plant cultivation under saline conditions.

It is important to acknowledge a key limitation of this study: the absence of a soluble zinc control (e.g., ZnSO_4_). This design precludes a definitive distinction between the effects attributable to the nanoparticle form of ZnO and those resulting from the nutritional supply of Zn²^+^ ions. Similar limitations are common in nanomaterial-plant interaction studies. Our findings therefore reflect the integrated response of *G. uralensis* to ZnO NPs treatment, which likely encompasses both nano-specific interactions and ionic effects. The observed modulation of phenylpropanoid and flavonoid pathways, along with ion homeostasis, could be driven by either or both mechanisms. Future studies incorporating comparative treatments with ionic zinc sources, coupled with analyses of ZnO NPs dissolution dynamics in the rhizosphere, are essential to disentangle these contributions and elucidate the precise mechanistic role of the nanoparticle itself.

## 5 Conclusion

In summary, this study demonstrates that ZnO nanoparticles can alleviate salt-induced growth inhibition and physiological disturbance in *Glycyrrhiza uralensis*. Integrated physiological, transcriptomic, and metabolomic analyses suggest that this mitigation is associated with adjustments in antioxidant-related processes and secondary metabolic pathways, particularly phenylpropanoid and flavonoid metabolism. While the specific contribution of nanoparticle versus ionic zinc requires further clarification, these findings provide mechanistic insights into ZnO NPs–mediated stress responses and support the potential application of nanomaterials in improving root-associated stress adaptation under saline conditions.

## Data Availability

The datasets presented in this study can be found in online repositories. The names of the repository/repositories and accession number(s) can be found in the article/**Supplementary Material**.
