# Supplementary material for: Integrated transcriptomic and metabolomic analysis reveals zinc oxide nanoparticles associated modulation of salt stress responses in Glycyrrhiza uralensis
Source: Front Plant Sci. 2026 Mar 3;17:1767899. doi: 10.3389/fpls.2026.1767899 (PMC12992307; doi:10.3389/fpls.2026.1767899)
Supplement: Supplementary file 1 [file Image1.pdf]

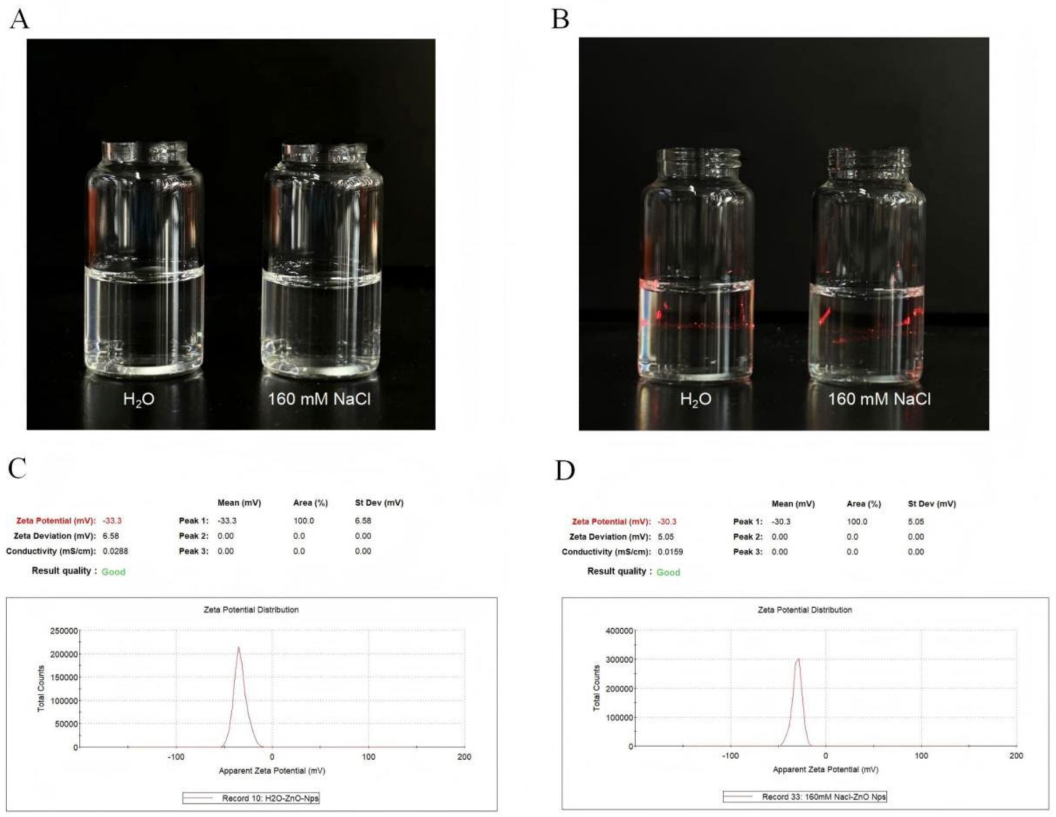


**Supplementary Fig. S1.** Assessment of the colloidal stability of ZnO NPs. Photographs of ZnO NPs dispersions in tap water and 160 mM NaCl solution after 24-hour standing. A, Dispersions without laser irradiation. B, The same dispersions irradiated with a 650 nm red laser pointer in a dark background. C, Zeta potential value in tap water. D, Zeta potential value in 160 mM NaCl solution.

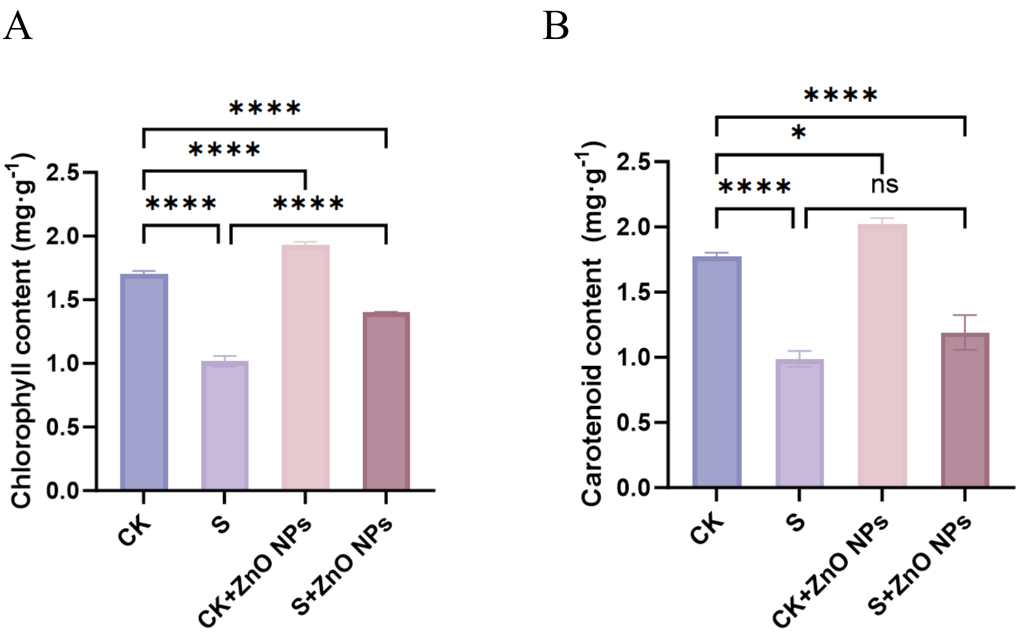


**Supplementary Fig. S2.** A, The content of Chlorophyll content of leaves under each treatment. B, The content of Carotenoid content of leaves under each treatment.

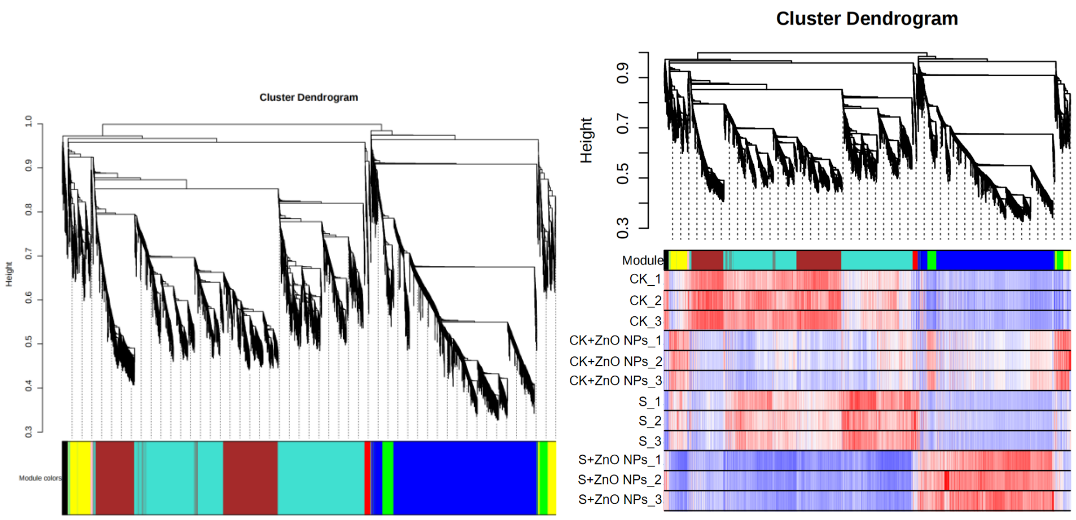


**Supplementary Fig. S3.** Nine modules were obtained among differentially expressed genes (DEGs) between the different treatments.

A

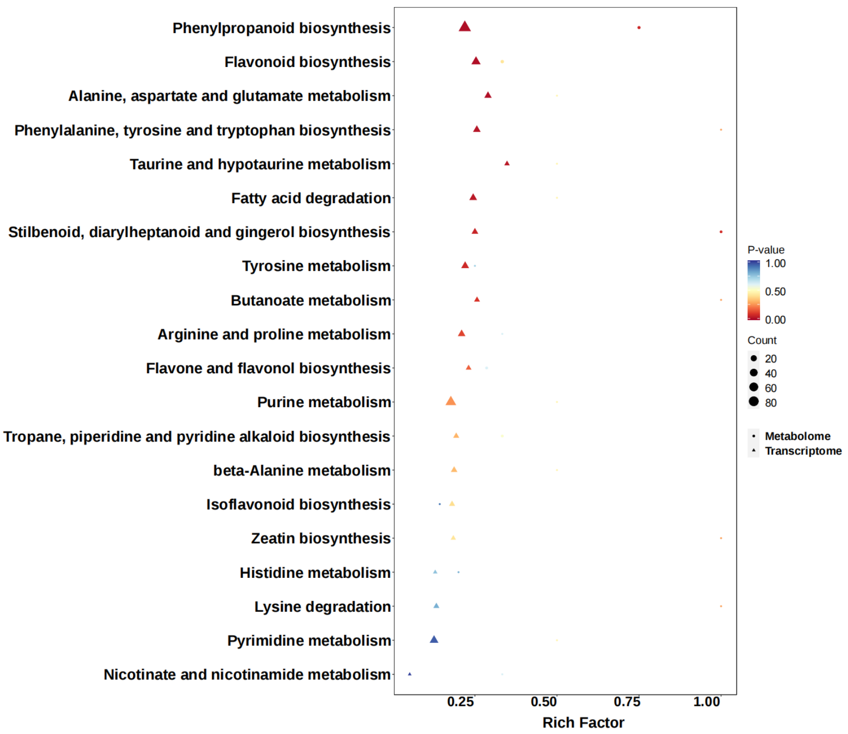
Phenylpropanoid biosynthesis
Flavonoid biosynthesis
Alanine, aspartate and glutamate metabolism
Phenylalanine, tyrosine and tryptophan biosynthesis
Taurine and hypotaurine metabolism
Fatty acid degradation
Stilbenoid, diarylheptanoid and gingerol biosynthesis
Tyrosine metabolism
Butanoate metabolism
Arginine and proline metabolism
Flavone and flavonol biosynthesis
Purine metabolism
Tropane, piperidine and pyridine alkaloid biosynthesis
beta-Alanine metabolism
Isoflavonoid biosynthesis
Zeatin biosynthesis
Histidine metabolism
Lysine degradation
Pyrimidine metabolism
Nicotinate and nicotinamide metabolism
0.25
0.50
0.75
1.00
Rich Factor
P-value
1.00
0.50
0.00
Count
20
40
60
80
Metabolome
Transcriptome

B

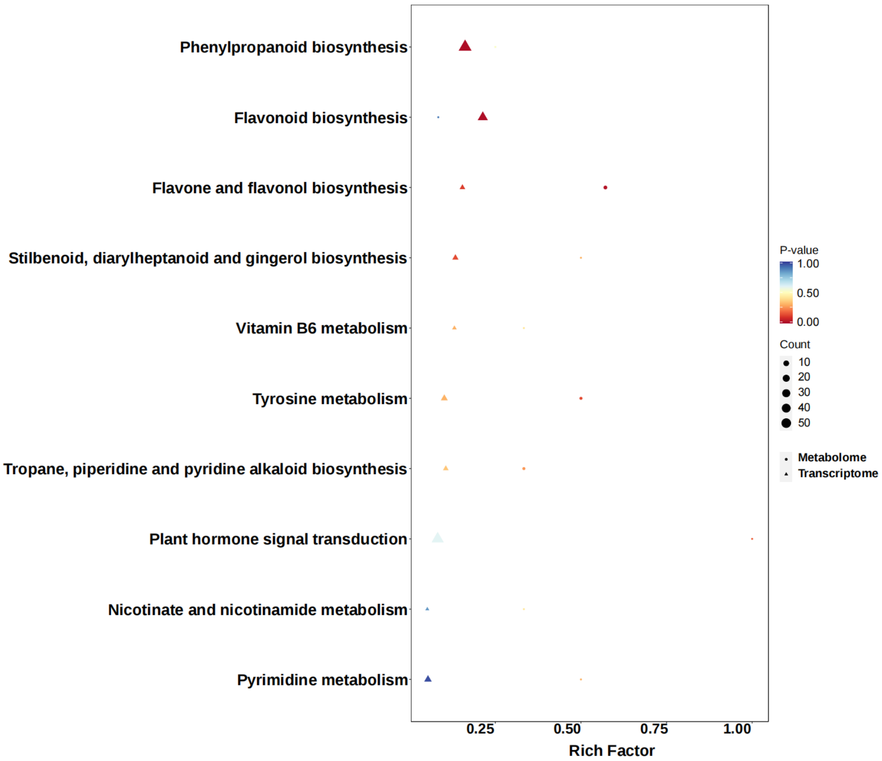
Phenylpropanoid biosynthesis
Flavonoid biosynthesis
Flavone and flavonol biosynthesis
Stilbenoid, diarylheptanoid and gingerol biosynthesis
Vitamin B6 metabolism
Tyrosine metabolism
Tropane, piperidine and pyridine alkaloid biosynthesis
Plant hormone signal transduction
Nicotinate and nicotinamide metabolism
Pyrimidine metabolism
0.25
0.50
0.75
1.00
Rich Factor
P-value
1.00
0.50
0.00
Count
10
20
30
40
50
Metabolome
Transcriptome

C

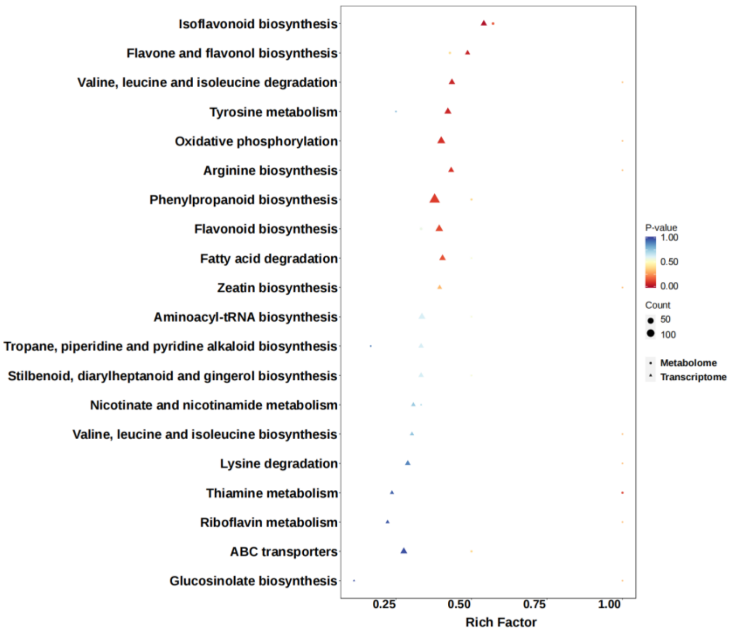


D

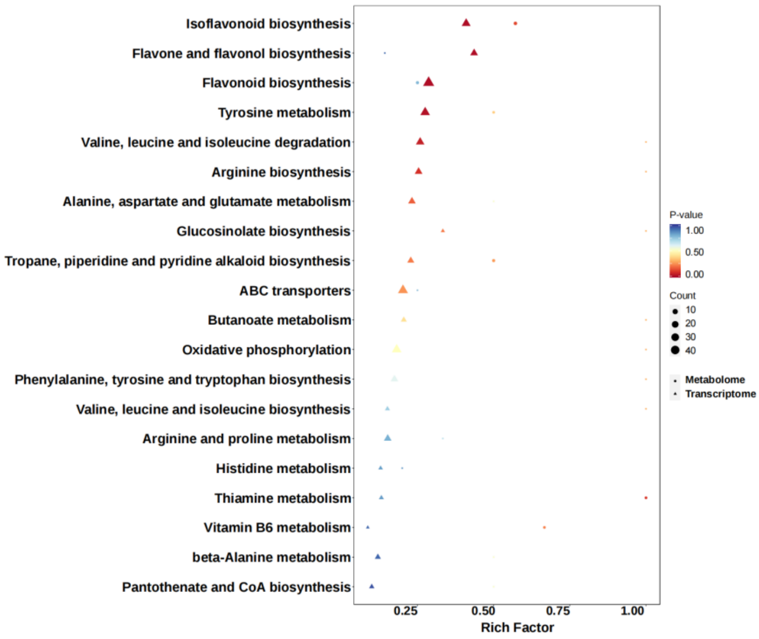


**Supplementary Fig. S3.** A, CK vs S differential metabolite and differential gene co enrichment bubble plot. B, CK vs CK+ZnO NPs differential metabolite and differential gene co enrichment bubble plot. C, CK vs M+ZnO NPs differential metabolite and differential gene co enrichment bubble plot. C, M vs M+ZnO NPs differential metabolite and differential gene co enrichment bubble plot.
